# Subaortic Membrane and Cardiac Catheterization—Beware of Diagnostic Pitfall

**DOI:** 10.1016/j.jscai.2024.102284

**Published:** 2024-10-29

**Authors:** Jonathan X. Fang, Georgi Fram, Dee Dee Wang, Pedro A. Villablanca, Brian P. O’Neill, Tiberio M. Frisoli, Gennaro Giustino, James C. Lee, William W. O’Neill, Pedro Engel Gonzalez

**Affiliations:** Center for Structural Heart Disease, Henry Ford Health System, Detroit, Michigan

**Keywords:** cardiac catheterization, cardiac hemodynamics, subaortic membrane, subaortic stenosis, transesophageal echocardiography

## Introduction

Left ventricular outflow obstruction can be valvular, supravalvular, or subvalvular; however, subaortic membrane is a rare cause of subvalvular obstruction.

## Case report

**Figure 1:**
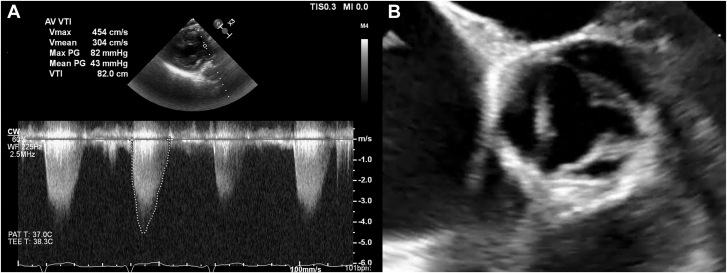
(**A**) Gradient across aortic valve and LVOT on transthoracic echocardiogram. (**B**) Normal aortic valve opening.

**Figure 2:**
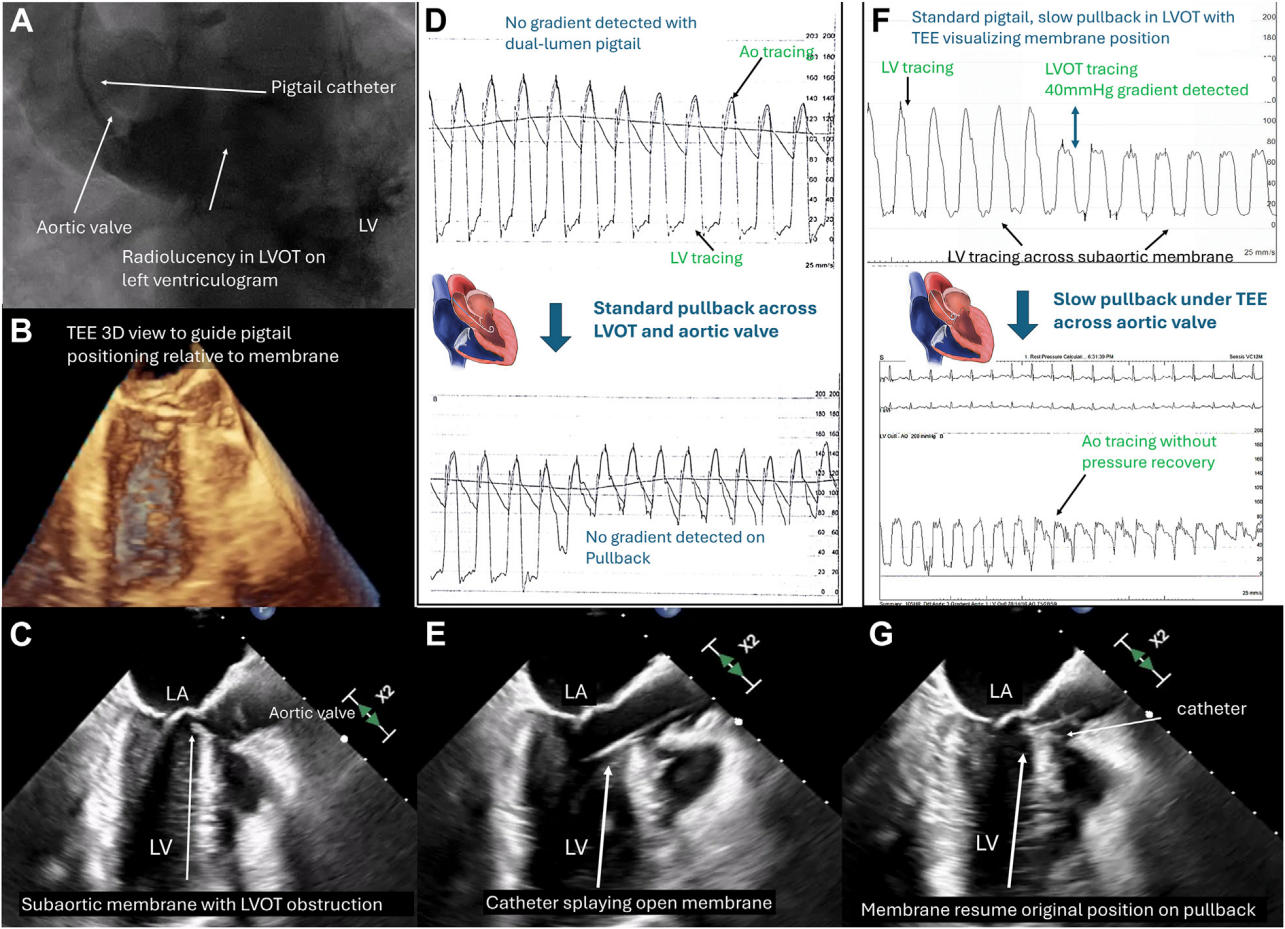
(**A-C**) Appearance of subaortic membrane on left ventriculogram and transesophageal echocardiography (TEE). (**D-E**) No gradient detected with a dual-lumen pigtail catheter as the subaortic membrane is splayed open as seen on TEE. (**F-G**) Slow pullback on TEE unmasks the pressure gradient as membrane resumes original position. LA, left atrium; LV, left ventricle; LVOT, left ventricular outflow tract.

A 39-year-old woman with a body mass index of 45 kg/m^2^ and hypertension presented with dyspnea with New York Heart Association class III symptoms. Physical examination revealed a faint systolic ejection murmur at the aortic area with no change in intensity on dynamic auscultation. A transthoracic echocardiogram showed poor echogenicity with a mean gradient of 43 mm Hg across the aortic valve and left ventricular outflow tract (LVOT) (Figure 1A) and significant ventricular hypertrophy. However, cardiac catheterization did not detect any pressure gradient across the LVOT and aortic valve with usage of a Langston dual-lumen pigtail catheter (Teleflex). A normal coronary arteriogram showed normal results, and multimodality imaging was limited by body habitus. We further investigated the discrepancy in the findings with simultaneous catheterization and transesophageal echocardiography (TEE). Aortic valve opening was normal (Figure 1B). Left ventriculogram revealed a faint radiolucency in the LVOT (Figure 2A), while TEE showed a discrete soft tissue ridge in the LVOT compatible with a subaortic membrane (Figure 2B, C). A dual-lumen pigtail catheter did not detect any pressure gradient—neither with simultaneous left ventricular and aortic pressure measurement nor with a pullback at standard speed (Figure 2D). Concurrent TEE showed that the membrane was splayed open by the catheter throughout the cardiac cycle with the presence of the catheter (Figure 2E). On pullback at normal speed, the membrane did not immediately resume its original position to cause LVOT obstruction. We switched to a standard (single-lumen) pigtail catheter and positioned it carefully under direction visualization, showing a pressure gradient of 40 mm Hg when the catheter was slowly dragged across the LVOT while allowing the membrane to resume its position causing LVOT obstruction. As the catheter was pulled back very slowly across the aortic valve, a change to aortic waveform without any pressure recovery was seen, signifying that the pressure gradient observed from true and not due to Bernoulli effect (Figure 2F-G). The patient was referred for surgery.

## Discussion

Cardiac catheterization, considered the gold standard, is often used to resolve discrepancy between imaging and other findings. Subaortic membranes are often challenging to diagnose, causing a gradient on echocardiogram despite a normal appearance of the aortic valve. The condition might be confused with hypertrophic cardiomyopathy on echocardiography alone and multimodality image might be necessary.^1^ The dual-lumen is a commonly used tool for assessing gradients and severity of valvular aortic stenosis. Dual-lumen catheters have been described to be able to accurately locate the level of obstruction when concomitant LVOT obstruction by subaortic membrane and hypertrophic cardiomyopathy is present.^2^ On the contrary, the concern of cardiac catheterization missing the diagnosis of subaortic membrane has also been reported.^3^ Our case illustrates the mechanism leading to missing of the diagnosis with standard cardiac catheterization methods alone, as well as a method of overcoming the diagnostic pitfall by the very slow pullback of a single-lumen pigtail catheter under TEE guidance. One other potential solution is the use of a pressure wire sent into the LVOT with the tip of a multipurpose catheter in the aortic root to obtain simultaneous aortic and LVOT tracing without disturbing the membrane position.^4^ Another potential solution is the simultaneous placement of a catheter through transeptal puncture in the left ventricle with another catheter in the aortic root, although this approach cannot pinpoint the anatomical level of obstruction. Overall, a high index of suspicion and a multimodality approach is required to reach the diagnosis of a subaortic membrane. The observation of a pressure gradient measurable by echocardiogram without the detection of any pressure gradient on cardiac catheterization should raise the suspicion of a subaortic membrane, warranting further scrutiny.

This study was performed in accordance with ethical principles of the tenets of the Declaration of Helsinki. Institutional board review approval for off-label use of investigative or therapeutic devices for diagnosis and management of structural heart disease has been obtained. Patient consent was obtained for the use of anonymized content for education purpose.

## Pearls in Hemodynamics

• A subaortic membrane can cause fixed subvalvular obstruction but can be splayed open by an LVOT catheter leading to the failure to detect an LVOT pressure gradient.
• The suspicion of a subaortic membrane should be raised when catheterization fails to detect a fixed left ventricular outflow pressure gradient that is evident on echocardiography.
• Potential solutions include pigtail catheter repositioning, the use of a pressure wire, or simultaneous transarterial and trans-septal access.
